# Arthroscopic debridement of the ankle in synovitis

**DOI:** 10.25122/jml-2023-0117

**Published:** 2023-08

**Authors:** Mohammad Jesan Khan, Naiyer Asif, Mohd Hadi Aziz, Siraj Ahmed Hasham Shaikh, Faiza Banu Siddiqui, Khwaja Moizuddin, Shibili Nuhmani

**Affiliations:** 1 Department of Orthopedic Surgery, Jawaharlal Nehru Medical College Hospital, Aligarh Muslim University, Aligarh, Uttar Pradesh, India; 2 Department of Anatomy, College of Medicine, Imam Abdulrahman Bin Faisal University, Dammam, Kingdom of Saudi Arabia; 3 Department of Physical Therapy, College of Applied Medical Sciences, Imam Abdulrahman Bin Faisal University, Dammam, Kingdom of Saudi Arabia

**Keywords:** Meislin's criterion, synovitis, arthroscopic debridement, synovial lining, AOFAS: American Orthopaedic Foot and Ankle Society, FADI: Foot and Ankle Disability Index, VAS: Visual Analogue Scale, NSAIDs: Nonsteroidal Anti-Inflammatory Drugs

## Abstract

Inflammation and hypertrophy of the ankle joint's synovial lining can occur due to various causes. Chronic pain and degenerative changes may be due to synovitis causing clinical manifestations through traction on the joint capsule. The failure of conservative treatment for at least six months indicates arthroscopic debridement, which can provide significant pain relief without the morbidity of extensive surgical exposures. This study was therefore conducted to establish the functional results of arthroscopic debridement of the ankle joint in synovitis. Fifteen patients with chronic ankle pain who had not responded to conservative treatment for approximately six months were included in the study. Arthroscopic debridement was performed using a shaver blade, followed by a postoperative ankle physiotherapy regimen. Patients were assessed preoperatively and postoperatively using the AOFAS, FADI, and VAS scores, with a mean follow-up period of 26 months. There was a significant improvement in the final clinical outcomes of the patients. The post-operative VAS score improved to 2.20±0.56 (2-4) (p-value=0.001), the AOFAS score was 86±8.25 (65-98) (p-value-0.001), and the FADI Score was 86.93±7.35(70-96) (p-value=0.001). Thirteen patients (86.67%) achieved outstanding or good results, while two had fair results, according to Meislin's criterion. One patient reported a superficial wound infection, which subsided with antibiotic therapy. The study findings indicate that arthroscopic ankle debridement is an efficient method to treat persistent ankle discomfort induced by synovitis, and it has a low postsurgical complications rate, quicker recovery, and less joint stiffness.

## INTRODUCTION

Inflammation and hypertrophy of the ankle joint's synovial lining can occur as a consequence of inflammatory arthritis, infection, and degenerative or neuropathic diseases. When chronic pain and degenerative changes are evident, it is important to keep in mind that synovitis may be present, which can cause clinical manifestations either directly or indirectly through traction on the capsule. Trauma and joint overuse can cause pain and swelling due to generalized inflammation of the joint synovium [1]. In most cases, conservative treatments lead to improvement.

Some patients experience continuous ankle discomfort and swelling without evidence of ankle instability. An arthroscopic examination occasionally reveals localized synovitis [2]. A clinical diagnosis can be established based on symptoms such as generalized ankle pain, effusion, and painful range of motion. It is essential to first rule out conditions like septic arthritis, gouty arthritis, and other systemic arthritis. Although there may be some signal alterations in magnetic resonance imaging (MRI), the diagnostic tests are generally negative. Restricted weight bearing, NSAIDs, and shoe modifications, including heel lift orthoses, ankle bracing, local injection, and physiotherapy, are the various treatment modalities available [1, 3]. When conservative treatment fails for at least 6 months, arthroscopic synovectomy is recommended, which offers considerable pain alleviation [4]. Arthroscopic irrigation and debridement use to treat infected ankle joints has also been described [5].

There is controversy regarding how to treat a patient who sustains an ankle injury and experiences prolonged symptoms despite a stable ankle joint. In recent years, due to advancements in small joint arthroscopy and the introduction of suitable instruments and scopes for smaller and tighter joints, arthroscopic debridement has gained popularity due to its minimally invasive nature, low morbidity, less joint stiffness, and faster recovery. Currently, ankle arthroscopy has been successfully used to treat various disorders, such as loose bodies, talar dome defects, degenerative disorders, and posttraumatic conditions [6-8]. However, there has been a paucity of data regarding the effect of arthroscopic debridement on ankle synovitis. This study was therefore conducted to establish the functional results of arthroscopic debridement of the ankle joint in synovitis.

## MATERIAL AND METHODS

This prospective study was conducted at the Department of Orthopedic Surgery from November 2019 to December 2022. It involved fifteen patients, including 10 males and 5 females, with a mean age of 38.80±15.68 years (ranging from 20 to 65 years). These patients experienced chronic ankle pain that did not respond to conservative treatments such as Nonsteroidal Anti-Inflammatory Drugs (NSAIDs) and repeated courses of physiotherapy for approximately 6 months. The exclusion criteria comprised patients with localized soft-tissue infection, tenuous vascular status, and ankle instability.

**Figure 1:**
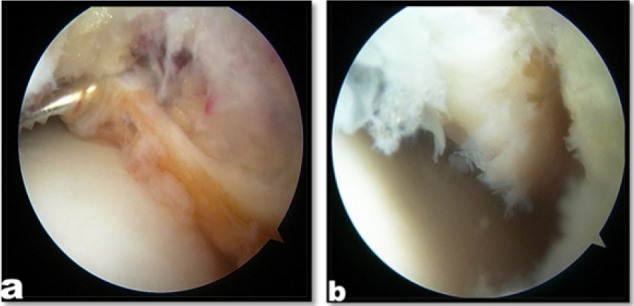
Arthroscopic view shows lateral gutter scar tissue, synovial hypertrophy (a), and lateral gutter after debridement (b)

**Figure 2:**
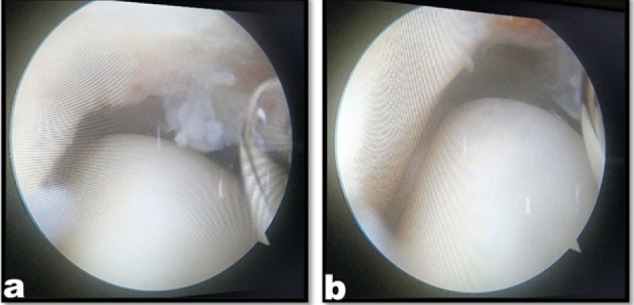
Medial gutter showing synovial hypertrophy (a), medial gutter after debridement (b)

A comprehensive personal and clinical history was obtained from each patient, followed by a thorough examination that included the Ankle Anterior Drawer test and Talar tilt test to assess ankle instability. To evaluate foot disability, we calculated the American Orthopaedic Foot and Ankle Society (AOFAS) Score and the Foot and Ankle Disability Index (FADI) score. In addition, the Visual Analogue Scale (VAS) score was calculated to document the severity of pain. All patients had persistent ankle pain with intermittent localized swelling. All ankles were stable on stability tests. An X-ray examination was done to assess the patient preoperatively. A senior surgeon performed all the operations, using a tourniquet in each case. The joint surfaces were carefully examined (Figures 1 and 2 A-B). The hypertrophic synovium was resected using a motorized shaver [9, 10], and the removed tissue was subjected to pathological investigation. Varus-valgus stresses and flexion-extension maneuvers aided in visualizing various areas of the ankle joint. On the second post-operative day, full weight-bearing was permitted as tolerable. Following this, they were discharged from the hospital. The post-operative physiotherapy program included a range of exercises such as proprioception, inversion/eversion exercises, isometric ankle strengthening, resistance ankle strengthening with Theraband, and range of motion exercises [11]. Patients’ functional outcomes were assessed using the AOFAS and the FADI scores, Meislin’s criteria, and the VAS score on subsequent follow-ups. We followed up with the patients for an average of 26 months (ranging from 18 to 32 months).

The study of Duan *et al*. [12] was used to calculate the sample size. He observed that the mean pre-operative and post-operative AOFAS scores were 38±12 and 85±7, respectively (p<0.01). Considering the values as a reference, the minimum sample size with 80% power of study and 5% level of significance was 12 patients. Data analysis was performed using SPSS software, version 20.0 (IBM Corp., Chicago). Wilcoxon Signed Rank Test was applied.

## RESULTS

The average age of the patients was 38.80±15.68, ranging from 20 to 65 years. 10 male and 5 female patients were included in our study (Table 1). These individuals were carefully monitored for an average duration of 26 months, ranging from 18 to 32 months. All of the patients had generalized synovitis. The synovial tissue was erythematous and inflamed, and the joint space could be seen after the initial synovectomy using a motorized shaver.

**Table 1:** Characteristics of the participants

Gender	
male	10
female	5
**Side involved**	
right	5 (33.33%)
left	10 (66.67%)
**Age (in years)**	38.80±15.68 (20-65)
**Follow-up period**	26 months (18-32)

Meislin’s criteria and the AOFAS, FADI, and VAS scores were calculated for each patient preoperatively. In the pre-operative group, 6 patients (40%) were in the “Fair” category and 9(60 %) were in the “Poor” category of AOFAS grading (Table 2). Eight patients (53.33 %) reported a “Moderate to severe” level of pain, and 7 patients (46.67 %) reported a “Very severe” level of pain on the VAS scale (Table 3). The pre-operative mean VAS score was 7±1.51 (5-9), the AOFAS score was 59.67±18.53 (24-79), and the FADI score was 48.53±18.82 (13-65) (Table 4).

**Table 2:** Grading of patients based on the pre-operative and post-operative AOFAS score

AOFAS grading	Pre-operative No. patients (%)	Post-operative No. patients (%)
Excellent (90-100)	0 (0%)	6 (40%)
Good (80-89)	0 (%)	7 (46.67%)
Fair (70-79)	6 (40%)	1 (6%)
Poor (<70)	9 (60%)	1 (6%)

**Table 3:** Grading of patients based on the pre-operative and post-operative VAS score

VAS Grading	No. patients (%)	No. patients (%)
No Pain(0)	0	0
Mild (1-3)	0	14 (93.33%)
Moderate To Severe (4-6)	8 (53.33%)	1 (6%)
Very Severe (7-9)	7 (46.67%)	0
Worst Pain Possible (10)	0	0

**Table 4:** Pre- and post-operative functional scores

	Pre-operative	Post-operative	p-value
AOFAS	59.67±18.53 (24 - 79)	86±8.25 (65 - 98)	0.001
VAS	7±1.51 (5 - 9)	2.20±0.56 (2 - 4)	0.001
FADI	48.53±18.82 (13-65)	86.93±7.35 (70 - 96)	0.001

Postoperatively, we did a final evaluation of the patients. There has been significant improvement in scoring and pain assessment. Six (40%) patients were in the “Excellent” category, 7(46.67%) patients were in the “Good” category, 1(6.67%) patient was in the “Fair” category, and 1(6.67 %) was in the “Poor” category of AOFAS grading (Table 2). Fourteen patients (93.33 %) reported a “Mild” level of pain, and 1 patient (6.67 %) reported a “Moderate to severe” level of pain on the VAS scale (Table 3). The post-operative VAS score improved, and it was 2.20±0.56 (ranging from 2 to 4), with a p-value of 0.001. Similarly, the AOFAS and FADI scores also improved, averaging 86±8.25 (65-98) and 86.93±7.35 (70-96), p-value 0.001, respectively (Table 4). Thirteen patients (86.67%) achieved outstanding or good results, while two had a fair result, according to Meislin's criterion. No patient had poor results (Table 5). One patient reported a superficial wound infection, which subsided with antibiotic therapy. No neurovascular complications were reported.

**Table 5:** Final assessment of patients based on Meislin’s criteria

Meislin’s criteria rating	No. of Patients (%)
Excellent (90-100)	7 (46.67%)
Good (80-89)	6 (40%)
Fair (70-79)	2 (13.33%)
Poor (<70)	0 (0%)

## DISCUSSION

Ankle arthroscopy has gained popularity as a diagnostic and therapeutic procedure during the last decade. Compared to other major joints like the knee and shoulder, ankle arthroscopy is still in its early stages. All intra-articular structures of the ankle can be directly seen with ankle arthroscopy without requiring an arthrotomy or malleolar osteotomy. The capacity to perform ankle diagnostic and surgical arthroscopy has improved due to technological advancements and a complete understanding of anatomy. It is a more desirable procedure than open arthrotomy due to the lower morbidity and quicker recovery time. The ankle joint of the patients we evaluated showed superfluous and often inflamed synovial tissue. The cause of synovitis in these patients was unknown and might be multifactorial. Most of them previously suffered an ankle sprain. It may have arisen as a result of an acute event or repeated ankle sprains irritating the joint. Hemarthrosis resulting in an acute inflammatory response could proceed to chronic synovitis with repeated joint irritation if previous injuries were present. They did not show any signs of ligamentous instability. In 86.67% of our patients, arthroscopic debridement resulted in symptom alleviation.

Ankle arthroscopy was suggested by Ogilvie-Harris, Gilbart, and Chorney [4] for patients experiencing pain even after six months of an ankle sprain. In a survey of more than 100 ankle arthroscopies, Martin *et al*. [13] found that patients with synovitis had the highest overall results (77% good or excellent). Conversely, individuals with degenerative joint conditions did not experience similarly positive results. Thein *et al*. [2] reported similar results in a study on sports-related synovitis. Barber *et al*. [14] suggested that an ankle sprain not responding to conservative therapy was a potential candidate for arthroscopic debridement. Cerulli *et al*. [15] discovered hypertrophic synovitis of the ankle in 24 out of 30 young individuals. The majority of the chronic lesions in these cases were posttraumatic in nature. In terms of etiology, the synovitis shown may have arisen as a result of an acute event or repeated ankle sprains irritating the joint.

Ahn *et al*. [16] performed arthroscopic synovectomy for synovitis in 31 patients. The average pre-operative and post-operative AOFAS ankle-hindfoot scores were 69 and 89, respectively. Tahir *et al*. [17] did an arthroscopic treatment of posterior ankle pain. The AOFAS score increased from 48.7 to 83.2 in patients with Flexor Hallucis Longus (FHL) tenosynovitis. FHL tenosynovitis usually coexists with os trigonum syndrome. In addition to os trigonum excision, hindfoot arthroscopy allows simultaneous treatment of FHL tenosynovitis, as well as debridement of the local synovitis. In patients with peroneal tenosynovitis, AOFAS increased from 60.2 to 82.7.

In their study, Woo Jin Choi *et al*. [18] conducted an arthroscopic synovectomy of the ankle in 18 patients diagnosed with rheumatoid arthritis. The patients had a mean age of 51 years, ranging from 28 to 82 [18]. They observed that the average AOFAS score increased from 65.2 before surgery to 85.7 at the final follow-up evaluation (p<.0001). Manuel Bondi *et al*. [19] conducted a study involving 42 patients, 24 men and 18 women, who underwent ankle arthroscopy for chronic ankle pain. The success of the procedure was rated as excellent or very good by 35 patients (83.3%). At the final follow-up, the mean AOFAS score was 96.09 (89-98). In a separate study by Duan *et al*. [12], 15 patients with chronic synovitis of the ankle underwent arthroscopic debridement. There were significant differences in VAS and AOFAS scores before and after treatment (p<0.01). In this study, the mean pre-operative AOFAS score was 59.67±18.53 (24-79). The low pre-operative AOFAS score in our patients might be due to the late presentation of the patients at an advanced stage of their condition. However, postoperatively, the mean AOFAS improved to 86±8.25 (ranging from 65 to 98) and was comparable to other studies.

In our study, the mean pre-operative FADI score was 48.53±18.82 (13-65), and the mean post-operative score was 86.93±7.35 (70-96).

In a study by Woo Jin Choi *et al*. [18], where arthroscopic synovectomy of the ankle was performed on 18 patients with rheumatoid arthritis, they observed that the symptoms of synovitis disappeared. The VAS score for pain also dropped from 5.6 points (range, 4-6 points) preoperatively to 2.2 points (range, 0-4 points) at the last follow-up. Similarly, Manuel Bondi *et al*. [19] investigated 42 patients (24 men and 18 women) who underwent ankle arthroscopy for chronic ankle pain, and the average VAS score for pain was 0.76 at the final follow-up. In our study, the mean pre-operative VAS score was 7±1.51 (ranging from 5 to 9), and the mean postoperative VAS score improved to 2.20±0.56 (2-4), comparable to other studies.

In a study conducted by Hassan *et al*. [20] involving 23 patients, they reported that 91% of the patients achieved good to excellent results. Similarly, Meislin *et al*. [21] found 90% of their 29 patients achieved good to excellent results, Ferkel *et al*. [22] reported 85% in their study of 31 patients, and Liu *et al*. [23] reported 87% in their study of 55 patients. Brennan *et al*. [11] also reported 83% good to excellent results. In our study, 13 patients (86.67 %) achieved excellent or good results, while 2 had a fair result, according to Meislin's criterion. This is more favorable than the 66% satisfaction rate recorded in open surgery [24] and comparable to other studies where arthroscopic intervention was done. Other studies have identified factors that indicate a poor outcome, including the absence of a pre-operative diagnosis, repeated inversion injuries, and chondral damage to the talus [25]. Fair results in two of our patients may have been caused by a lack of definitive pre-operative diagnosis or inadequate synovectomy.

Common complications reported during ankle arthroscopy in other studies are superficial peroneal nerve injury during anterolateral portal placement [26] and sural nerve injury during posterolateral portal placement [27]. Injury to the Flexor hallucis longus tendon, posterior tibial artery, and tibial nerve may occur during posteromedial portal placement [28]. Articular cartilage damage may be the most commonly unreported consequence of arthroscopy of any joint [28]. However, none of our patients reported any such complication. This study also supports the findings of other studies that highlight the advantages of the arthroscopic technique, such as reduced morbidity, less invasiveness, minimal blood loss, and better visualization of intraarticular pathology [29, 30]. This study has several limitations, including a relatively small sample size and a relatively short average follow-up period.

## CONCLUSION

Finally, we believe that arthroscopic ankle debridement can successfully treat persistent ankle discomfort induced by synovitis, and it has a low postsurgical complications rate, quicker recovery, and less joint stiffness.

## Data Availability

The data associated with the paper are not publicly accessible but are available from the corresponding author upon reasonable request.

## Personal thanks

The author would like to thank all the participants of the study.
